# Good manufacturing practice-compliant isolation and culture of human umbilical cord blood-derived mesenchymal stem cells

**DOI:** 10.1186/1479-5876-12-56

**Published:** 2014-02-24

**Authors:** Phuc Van Pham, Ngoc Bich Vu, Vuong Minh Pham, Nhung Hai Truong, Truc Le-Buu Pham, Loan Thi-Tung Dang, Tam Thanh Nguyen, Anh Nguyen-Tu Bui, Ngoc Kim Phan

**Affiliations:** 1 Laboratory of Stem Cell Research and Application, University of Science, Vietnam National University, Ho Chi Minh city, Vietnam

**Keywords:** Mesenchymal stem cells, Platelet-rich plasma, Umbilical cord blood, Good manufacturing practice, Clinical application

## Abstract

**Background:**

Mesenchymal stem cells (MSCs) are an attractive source of stem cells for clinical applications. These cells exhibit a multilineage differentiation potential and strong capacity for immune modulation. Thus, MSCs are widely used in cell therapy, tissue engineering, and immunotherapy. Because of important advantages, umbilical cord blood-derived MSCs (UCB-MSCs) have attracted interest for some time. However, the applications of UCB-MSCs are limited by the small number of recoverable UCB-MSCs and fetal bovine serum (FBS)-dependent expansion methods. Hence, this study aimed to establish a xenogenic and allogeneic supplement-free expansion protocol.

**Methods:**

UCB was collected to prepare activated platelet-rich plasma (aPRP) and mononuclear cells (MNCs). aPRP was applied as a supplement in Iscove modified Dulbecco medium (IMDM) together with antibiotics. MNCs were cultured in complete IMDM with four concentrations of aPRP (2, 5, 7, or 10%) or 10% FBS as the control. The efficiency of the protocols was evaluated in terms of the number of adherent cells and their expansion, the percentage of successfully isolated cells in the primary culture, surface marker expression, and in vitro differentiation potential following expansion.

**Results:**

The results showed that primary cultures with complete medium containing 10% aPRP exhibited the highest success, whereas expansion in complete medium containing 5% aPRP was suitable. UCB-MSCs isolated using this protocol maintained their immunophenotypes, multilineage differentiation potential, and did not form tumors when injected at a high dose into athymic nude mice.

**Conclusion:**

This technique provides a method to obtain UCB-MSCs compliant with good manufacturing practices for clinical application.

## Introduction

Mesenchymal stem cells (MSCs) are one of the most studied and applied types of stem cells to date. These cells were first described by Friedenstein et al. as a cell population similar to fibroblasts [1], which can differentiate into multiple cell types such as osteoblasts, adipocytes, and chondrocytes [2]. MSCs have been isolated from many tissues including bone marrow [3,4], adipose tissue [5-7], peripheral blood, umbilical cord blood (UCB) [8-10], banked UCB [11-14], umbilical cords [15,16], placenta [17], amniotic fluid [18], dental pulp [19], and menstrual blood [20].

Compared with other stem cell sources, UCB-MSCs have advantages such as non-invasive recovery, the abundance of MSCs, and well-known characteristics. In both pre-clinical and clinical settings, MSCs have been studied to treat a various diseases. Pre-clinically, UCB-MSCs have been used to treat neonatal brain injury [21], fibrocartilaginous embolic myelopathy [22], spinal cord injury [23,24], diabetic renal injury [25,26], bone loss [27], ischemia [28,29], hearing loss [30], damaged corneal endothelium [31], Alzheimer’s disease [32], graft-versus-host disease (GVHD) [33], acute hepatic necrosis [34], diabetes mellitus [35], and liver cirrhosis [36]. Clinically, UCB-MSCs have been transplanted for treatment of autism [37], hereditary spinocerebellar ataxia [38], foot disease in patients with type 2 diabetes mellitus [39], and basilar artery dissection [40]. Clinical trials (retrieved from clinicaltrial.gov) include mesenchymal stem cell transplantation for engraftment of unrelated hematopoietic stem cell transplantation (NCT00823316), treatment of steroid-refractory acute or GVHD (NCT01549665), articular cartilage defect treatment (NCT01733186), and hematologic malignancy treatment (NCT01854567).

The main concern in UCB-MSC applications is *in vitro* expansion that is mostly affected by the culture medium. For production protocols of UCB-MSCs under clinical conditions, it is essential to include sterility controls, analysis for viral markers, and genetic testing such as karyotyping. Currently, UCB-MSCs can be produced at a GMP (good manufacturing practice) grade by automated processing protocols and some novel protocols. Procedures have been developed to isolate mononuclear cells (MNCs) in closed systems such as the SEPAX device [41,42]. Other systems can also be used to expand MSCs such as the Cell Stack System [43]. However, almost all of these methods require fetal bovine serum (FBS) for culture. FBS-based medium has some limitations associated with clinical application, especially prion and viral transmission or adverse immunological reactions against xenogenic components.

Some novel methods use human serum for MSC culture, especially platelet-rich plasma (PRP). Recent studies have used PRP from peripheral blood [44-48] and UCB [49-52], which showed that PRP from peripheral blood or UCB significantly stimulates the proliferation of MSC from bone marrow [45,50], UCB [49,53], or adipose tissue [44,54]. More importantly, MSCs cultured in medium supplemented with PRP exhibit a normal phenotype and characteristics [49-52], and maintain their multipotency for differentiation into adipocytes, osteoblasts, and chondrocytes. Taken together, these studies show that PRP can replace FBS for *in vitro* MSC expansion.

All of these previous protocols have used allogeneic PRP. The use of PRP allows MSCs to avoid xenogenic immunological reactions, and prion and viral transmission, but MSCs may encounter human viral transmission and immunological reactions induced by allogeneic components. According to the European Medicines Agency and regulation No. [EC] 1394/2007 of the European Commission, MSC are considered as medicinal products [55] and must be produced in compliance with GMP. The GMP standards ensure that cells are produced with the highest standards of sterility, quality control, and documentation following a standard operating procedure. Therefore, in this study, we aimed to establish an UCB-MSC isolation protocol using autologous PRP from the same umbilical blood sample. This protocol is GMP compliant and can be used for clinical applications.

## Materials and methods

### UCB collection and sample selection for study

UCB was collected from the umbilical cord vein with informed consent of the mother. The collection was performed in accordance with the ethical standards of the local ethics committee. To eliminate differences between UCB samples, the stem cell quantity was enumerated based on the number of hematopoietic stem cells (HSCs) using an Enumeration Pro-Count Kit (BD Bioscience) following the manufacturer’s guidelines. Only samples with ≥1 × 10^6^ HSCs/ml were used in experiments.

### MNC isolation and activated PRP preparation

First, blood samples were centrifuged at 2000 rpm for 15 min. The cell pellet was kept to isolate MNCs and the plasma was collected and centrifuged at 3500 rpm for 10 min. To prepare activated PRP (aPRP), a third of the plasma volume and the platelet pellet was collected and resuspended, and then 100 μL CaCl_2_ per 1 mL of PRP was added to activate growth factor release. The samples were then incubated at 37°C for 30 min or until the occurrence of clotting. The centrifuged blood cells were diluted at a ratio of 1:1 with phosphate buffered solution (PBS) and then applied to density centrifugation using Ficoll Hypaque (1.077 g/mL; Sigma-Aldrich, St Louis, MO). The collected MNCs were washed twice with PBS and then applied to experiments.

### Primary culture

Twenty UCB samples were used for primary culture. MNCs were cultured in Iscove modified Dulbecco medium (IMDM) containing 1% antibiotic-mycotic (Sigma-Aldrich, Louis St, MO), 10 ng/mL epidermal growth factor (EGF), 10 ng/mL basic fibroblast growth factor (bFGF), and various concentrations of aPRP (2, 5, 7, or 10%) or 10% fetal bovine serum (FBS) for the control. The cells were plated at 5 × 10^4^cells/mL in T-75 flasks (Corning) and incubated at 37°C with 5% CO_2_. After 3 days of incubation, 6 mL of fresh media were added to each flask. After 7 days, the media were replaced with fresh media. Then, the media was replaced every 4 days until the cells reached 70–80% confluence. The efficiency of the media was evaluated by the time required for adherent cells to appear and then reach 70–80% confluence for the first subculture.

### Secondary culture

After successful primary culture, the samples were subcultured to evaluate the effects of the various media. The proliferation rate was evaluated by the eXCELLIgence system (Roche Applied Science, Indianapolis, IN). A total of 1 × 10^3^ cells were seeded into each well of a 96-well E-plate in triplicate. The culture plates were placed into the eXCELLIgence system and incubated at 37°C with 5% CO_2_. Cell proliferation was monitored for 300 h with fresh medium changes every third day. Both the cell doubling time and slope value were determined by the software of the eXCELLIgence system.

### Flow cytometry

Cell markers were analyzed following a previously published protocol [11]. Briefly, cells were washed twice in PBS containing 1% bovine serum albumin (Sigma-Aldrich). The cells were then stained with anti-CD13-FITC, anti-CD14-FITC, anti-CD34-FITC, anti-CD44-PE, anti-CD45-FITC, anti-CD73-FITC, anti-CD90-PE, anti-CD105-FITC, anti-CD106-PE, anti-CD166-PE, or anti-HLA-DR-FITC antibodies (all purchased from BD Biosciences, San Jose, CA). Stained cells were analyzed by a FACSCalibur flow cytometer (BD Biosciences). Isotype controls were used in all analyses.

### In vitro differentiation

For differentiation into adipogenic cells, UCB-MSCs were differentiated as described previously [9]. Briefly, passage 5 cells were plated at 1 × 10^4^ cells/well in 24-well plates. At 70% confluence, the cells were cultured for 21 days in IMDM containing 0.5 mmol/L 3-isobutyl-1-methyl-xanthine, 1 nmol/L dexamethasone, 0.1 mmol/L indo-methacin, and 10% FBS (all purchased from Sigma-Aldrich). Adipogenic differentiation was evaluated by observing lipid droplets in cells under a microscope.

For differentiation into osteogenic cells, UCB-MSCs were plated at 1 × 10^4^ cells/well in 24-well plates. At 70% confluence, the cells were cultured for 21 days in IMDM containing 10% FBS, 10^-7^ mol/L dexamethasone, 50 μmol/L ascorbic acid-2 phosphate, and 10 mmol/L β-glycerol phosphate (all purchased from Sigma-Aldrich) [9]. Osteogenic differentiation was confirmed by Alizarin red staining.

For differentiation into chondrogenic cells, UCB-MSCs were induced to differentiate by a commercial medium for chondrogenesis (StemPro Chondrogenesis Differentiation Kit, A10071-01; Life Technologies). UCB-MSCs were differentiated in pellet form according to the manufacturer’s guidelines. After 21 days, the cell pellets were stained with an anti-aggrecan monoclonal antibody (BD Biosciences).

### Tumorigenicity assay

The tumorigenicity of UCB-MSCs was examined in athymic nude mice. All manipulations of mice were approved by the Local Ethics Committee of Stem Cell Research and Application, University of Science (Ho Chi Minh city, Vietnam). Each mouse was injected subcutaneously with 5 × 10^6^ cells (three mice per group). As a positive control, the mice were also injected with breast cancer cells at a different site. Tumor formation in mice was followed up for 3 months.

### Statistical analysis

The significance of differences between mean values was assessed by t-tests and analysis of variance. A P-value of less than 0.05 was considered to be significant. All data were analyzed by Prism 6 software.

## Results

### Primary cell culture

**Figure 1:**
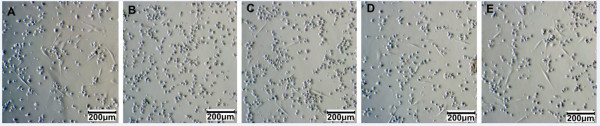
**UCB-MSC candidates adhered and exhibited a particular shape.** After 72 h of culture, adherent UCB-MSC candidates appeared in all media. However, the highest numbers of cells appeared in 10% FBS **(A)** and 10% aPRP **(E)**, and the number of adherent cells gradually decreased in 7% **(D)**, 5% **(C)** and 2% aPRP **(B)**.

**Figure 2:**
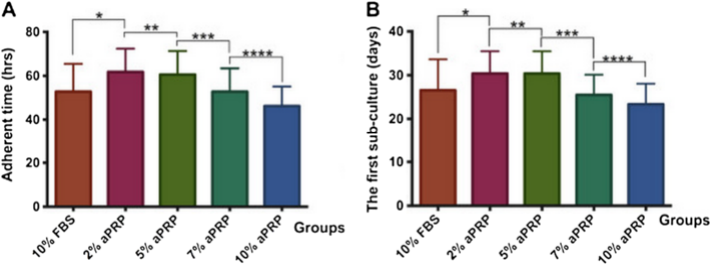
**Timing of adherence and confluence of primary cultured cells.** In 10% aPRP, MNCs adhered the soonest, and the adherence time gradually increased as the concentration of aPRP was decreased gradually **(A)**. Consequently, the time needed to reach 70–80% confluence in 10% aPRP was the shortest, which gradually increased as the concentration of aPRP decreased gradually **(B)**.

We collected 30 UCB samples of which 20 were applied to experiments. For primary culture, MNCs from the same sample were divided and cultured in five different media containing 10% FBS or 2, 5, 7, or 10% aPRP. There were differences in the time needed for MNCs to adhere and exhibit a particular shape in the various media. For example, at 72 h post-plating, there were clear differences in the number of adhered and fibroblast-like cells (Figure 1). The trend among the various media indicated that the number of cells gradually increased in 2, 5, 7, or 10% aPRP or 10% FBS in that order. The data from the 20 samples are presented in Figure 2.

As shown in Figure 2A, fibroblast-like cells appeared the most rapidly in 10% aPRP (46.20 ± 8.94 h), which was significantly sooner than that in 7% aPRP (52.80 ± 10.59 h), 5% aPRP (60.60 ± 10.64 h), 2% aPRP (61.80 ± 10.50 h), and 10% FBS (52.80 ± 12.56 h). These results showed that increases of the aPRP concentration led to decreases in the time needed for MNCs to adhere and exhibit a fibroblastic shape, indicating that components of aPRP were important for adherence and proliferation of UCB-MSCs.

These times also dictated the number of days until the first subculture (Figure 2B). As a result, 70–80% confluence was reached at 23.35 ± 4.73 days in 10% aPRP, whereas 25.50 ± 4.62, 30.40 ± 5.05, 30.40 ± 5.05, and 26.55 ± 7.05 days were required in 7, 5, or 2% aPRP, or 10% FBS, respectively.

### Cell proliferation rates in the various media

**Figure 3:**
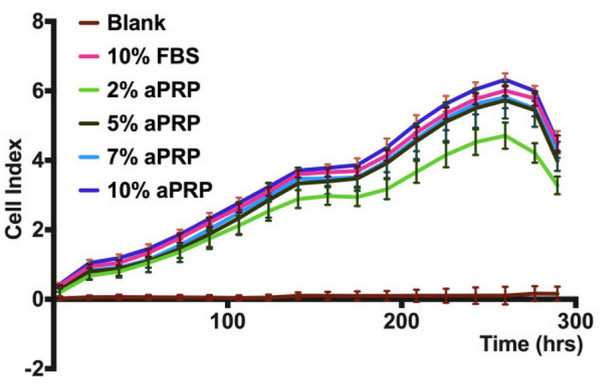
**Cell proliferation in the various media as recorded by the exCelligence method.** The results showed that the proliferation rates of cells in 5, 7, or 10% aPRP, or 10% FBS were not significantly different, while there was a significant difference in 2% aPRP.

After subculture, five samples were used to evaluate the effects of the various media on UCB-MSC proliferation. The results are presented in Figure 3. The proliferation rates of the cells in the various media were recorded from 0 to 300 h. At 0–130 h, the proliferation rates among the media were not significantly different. However, from 130 to 260 h, the proliferation rates were significantly different in 5, 7, or 10% aPRP, or 10% FBS compared with that in 2% aPRP. From 260 to 300 h, cells in all the various media underwent contact inhibition and death. As shown in Figure 3, the proliferation rate of cells in 10% aPRP was higher than that in the other media but the difference was not significant.

**Figure 4:**
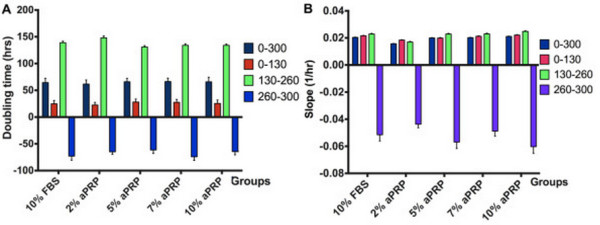
**Doubling times and slope values in the various media.** The values were obtained at three stages, 0–130 h, 130–260 h, and 260–300 h, and the whole proliferation curve at 0–300 h. There were no significant differences between the doubling times **(A)** and slope values **(B)** in 5, 7, or 10% aPRP, or 10% FBS, but a significant difference in 2% aPRP.

We also compared the cell doubling times and slope values (Figure 4). The doubling time is the time needed for the cell population to double in number. There were no differences in the doubling times for the whole period from 0 to 300 h. However, there were significant changes at each period from 0 to 130 h and 130 to 260 h. In the early stage (0–130 h), the doubling times were similar between the media (24.9 ± 6.11, 22.6 ± 4.9, 28.1 ± 5.71, 27.6 ± 5.52, and 25.5 ± 6.48 h in 10% FBS or 2, 5, 7, or 10% aPRP, respectively) (P < 0.05). In the next period (from 130 to 260 h), cells in all the various media proliferated with increasing doubling times. At this stage, the doubling times were similar in 5, 7, or 10% aPRP (P < 0.05) (131 ± 2.51, 134 ± 3, and 134 ± 2.49 h in 5, 7, or 10% aPRP, respectively). In contrast, the doubling time in 2% aPRP suddenly increased to 148 ± 3.63 h. In 10% FBS, the doubling time was 139 ± 2.82 h which was lower than that in 2% aPRP but higher than that in 5, 7, or 10% aPRP, but the difference was not significant.

These data further confirmed that the proliferation rate of UCB-MSCs in 5, 7, or 10% aPRP were similar to that in 10% FBS, but higher than that in 2% aPRP. The slope values also supported these results. In the early stage (0–130 h), there were no differences in the slope values among the media (0.021 ± 0.001, 0.018 ± 0.001, 0.020 ± 0.001, 0.021 ± 0.001, and 0.021 ± 0.001 for 10% FBS or 2, 5, 7, or 10% aPRP, respectively). In the next stage (130–260 h), the slope values exhibited significant differences between 10% FBS or 5, 7, or 10% aPRP (0.023 ± 0.001, 0.023 ± 0.001, 0.023 ± 0.001, and 0.024 ± 0.001, respectively) compared with that in 2% aPRP.

### Phenotypes of MSCs

**Figure 5:**
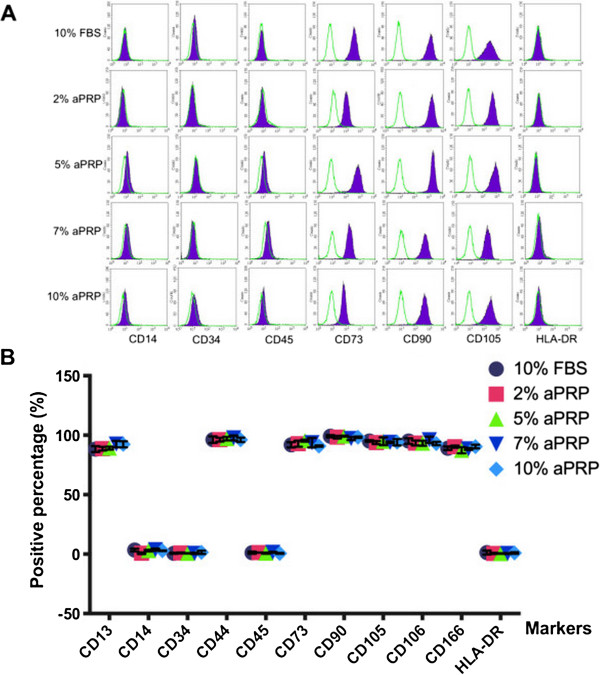
**Immunophenotypes of MSCs in the various media.** The results include flow cytometric analysis of CD13, CD14, CD34, CD44, CD45, CD73, CD90, CD105, CD106, CD166, and HLA-DR **(A)**. Data were analyzed and presented in a graph **(B)**.

MSC-specific marker expression of UCB-MSCs cultured in the various media were evaluated and compared as presented in Figure 5. The results showed that UCB-MSCs in all the media exhibited a MSC-specific marker profile, positivity for CD13, CD44, CD73, CD90, CD105, CD106, and CD166, and negativity for CD14, CD34, CD45, and HLA-DR. The expression levels of positive markers in MSCs were similar among the media.

**Figure 6:**
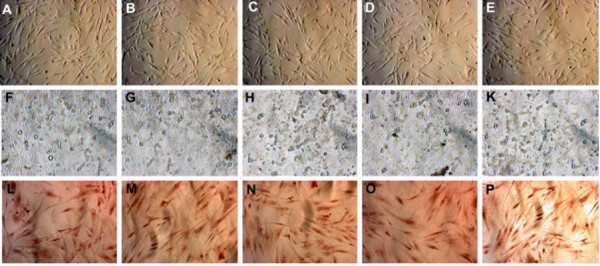
**MSCs cultured in the various media maintain their potential for differentiation into adipocytes and osteoblasts.** Compared with the control (**A**, **B**, **C**, **D**, and **E** are 10% FBS or 2, 5, 7, or 10% aPRP, respectively), MSCs accumulated lipid droplets in their cytoplasm after 21 days of induction (**F**, **G**, **H**, **I**, and **K** are 10% FBS or 2, 5, 7, or 10% aPRP, respectively). Following induction with osteoblast differentiation medium, the cells differentiated into osteoblasts that were positive for Alizarin red staining (**L**, **M**, **N**, **O**, and **P** are 10% FBS or 2, 5, 7, or 10% aPRP, respectively).

**Figure 7:**
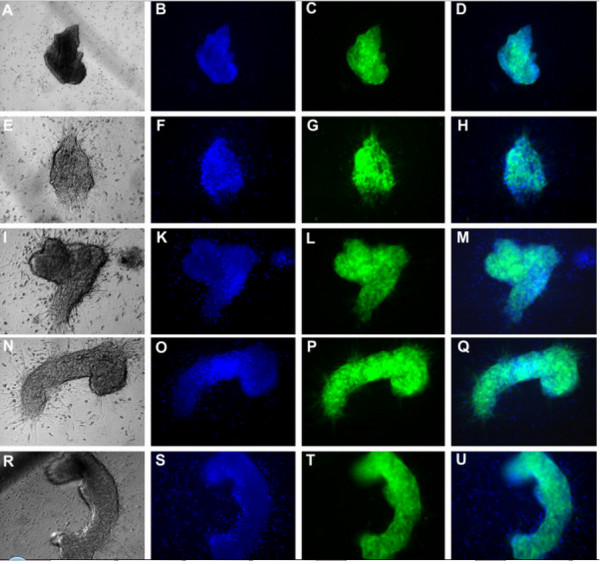
**MSCs cultured in the various media can differentiate into chondrocytes.** Differentiated cells (**A**, **E**, **I**, **R**, and **N** are 10% FBS or 2, 5, 7, or 10% aPRP, respectively) were stained with Hoescht 33342 **B**, **F**, **K**, **O**, and **S** are 10% FBS or 2, 5, 7, or 10% aPRP, respectively) and an anti-aggrecan monoclonal antibody (**C**, **G**, **L**, **P** and **T** are 10% FBS or 2, 5, 7, or 10% aPRP, respectively). Merged images with brightfield as shown in **D**, **H**, **M**, **Q**, and **U** for 10% FBS or 2, 5, 7, or 10% aPRP, respectively.

UCB-MSCs cultured in the various media were examined for their capacity to differentiate into adipogenic, osteogenic, and chondrogenic lineages. The results of differentiation assays are presented in Figures 6 and 7. In all media, the cells successfully differentiated into adipocytes, osteoblasts, and chondrocytes. Compared with cells prior to induction (Figure 6A–E), cells in all media accumulated lipid droplets in their cytoplasm after induction with adipocyte differentiation medium (Figure 6F–K). Following induction with osteoblast differentiation medium, cells in all media accumulated Ca^2+^ and Mg^2+^ in the cytoplasm and extracellular matrix, which were stained with Alizarin red (Figure 6I–P). After 21 days of chondrogenic differentiation, cells in all media expressed aggrecan, a cartilage-specific proteoglycan core protein (Figure 7).

### Tumorigenicity of UCB-MSCs

**Figure 8:**
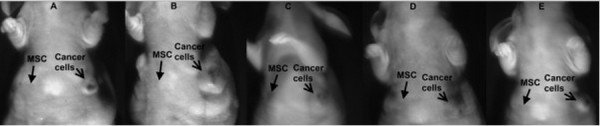
**Tumorigenicity of UCB-MSCs in athymic nude mice.** MSCs from all groups (10% FBS **(A)** or 2 **(B)**, 5 **(C)**, 7 **(D)**, or 10% aPRP **(E)**) could not cause tumors in the athymic nude mice while breast cancer cells easily caused tumors when injected in the same mice. MSCs were injected in the left breast; and breast cancer cells were injected in the left breast.

UCB-MSCs cultured in the various media were injected into athymic nude mice. Breast cancer cells were also injected at a different location as a positive control. Injection of UCB-MSCs resulted in no tumor formation, whereas injection of breast cancer cells resulted in tumor formation in all mice (Figure 8).

## Discussion

The aim of this study was to establish a GMP-compliant protocol for isolation of UCB-MSC for clinical application. Therefore, we eliminated xenogenic and allogeneic components that can cause immunological reactions and viral transmission. Our approach replaced FBS in the medium with autologous aPRP that was isolated from the same UCB sample used to isolate MNCs.

Because the source of autologous aPRP was limited and the necessary number of MSCs for clinical application is high, we evaluated four concentrations of aPRP in complete medium, including 2, 5, 7, and 10%, and 10% FBS as a control. The effects of aPRP on MSC proliferation was evaluated in primary and secondary cultures. In primary culture, medium containing 10% aPRP significantly stimulated MSC proliferation compared with that of the other aPRP concentrations and 10% FBS. In medium containing 10% aPRP, MSCs adhered quickly and proliferated rapidly. These observations demonstrated that aPRP contains all the essential components similar to those in FBS for support of cell attachment and proliferation. In fact, aPRP contains high amounts of attachment proteins such as fibrin, fibronectin, vitronectin, and thrombospondin [49,56]. In addition, aPRP contains several growth factors that stimulate cell proliferation, such as EGF, acidic fibroblast growth factor, keratinocyte growth factor, vascular endothelial growth factor, platelet-derived growth factor, hepatocyte growth factor, and bFGF [49,52,57]. Compared with bovine growth factors in FBS, aPRP can be obtained from humans, allowing better interactions between growth factors and cell receptors. In primary culture, the time needed to reach confluency in 10% aPRP indicated that this concentration of aPRP induced stronger MSC proliferation than that of FBS.

In expansion culture, the effects of the four concentrations of aPRP were also evaluated alongside the 10% FBS control. The results showed that there were no differences between 5, 7, or 10% aPRP supplementation compared with 10% FBS, but these aPRP concentrations showed significantly different effects than those of 2% aPRP. In fact, we confirmed these effects by the proliferation curve, doubling time, and slope value for proliferation. Based on proliferation curves, we could easily recognize differences in the proliferation rates between 2% aPRP and the other aPRP concentrations. However, the proliferation rates in 5, 7, or 10% aPRP or 10% FBS were not increased significantly. These data indicated the differences in the growth factor concentrations at 5, 7 and 10% aPRP did not cause any significant difference in the proliferation of UCB-MSCs.

Other important properties that we evaluated were the effects of aPRP-containing medium on surface marker expression and multilineage differentiation of UCB-MSCs. We used the marker profile of positive and negative markers suggested by Domicini et al. [58]. The results showed that UCB-MSCs maintained their marker expression in aPRP-containing media compared with that in medium supplemented with FBS. UCB-MSCs cultured in aPRP- and FBS-containing media did not express hematopoietic markers, such as CD14, CD34 and CD45, or HLA-DR, while they expressed stromal cell markers such as CD13, CD44, CD73, CD90, CD105, and CD106. These results completely agreed with other studies of UCB-MSCs cultured in FBS-supplemented medium [8,9,59-61], human peripheral blood-derived PRP [62], and UCB-derived PRP [49-51]. UCB-MSCs cultured in the various media also exhibited multilineage differentiation to adipocytes, osteoblasts, and chondrocytes. These results were similar to those of UCB-MSCs isolated in serum-supplemented medium [8,9,59-61].

Some previous studies have shown that PRP has some effects on MSCs. In addition to PRP strongly stimulating MSC proliferation, PRP also triggers differentiation. However, these effects of PRP are different between the various types of MSCs. PRP induces UCB-MSCs and bone marrow-derived MSCs to differentiate into osteoblasts [46], [53,63] and adipose-derived stem cells to differentiate into chondrocytes [7]. In this study, we did not evaluate the effects of PRP on UCB-MSC differentiation. However, we found that MSCs cultured in medium containing 2, 5, 7, or 10% aPRP maintained their potential for differentiation into adipocytes, osteoblasts, and chondrocytes. This result indicated that aPRP cultured UCB-MSCs had not become mature cells such as osteoblasts or chondrocytes. In fact, in previous studies, although MSCs have been proposed to differentiate into osteoblasts and chondrocytes, they also maintain their differentiation capacity for adipocytes, osteoblasts, and chondrocytes [62,64]. In our final analysis, UCB-MSCs cultured in the various media were examined for tumorigenicity in athymic nude mice. The results showed that all mice injected with UCB-MSCs cultured in the various media showed no tumor formation at the injection site, while cancer cells caused tumor formation in all mice at their injection site.

In summary, we successfully established a protocol for isolation of GMP-compliant UCB-MSCs. For primary culture, IMDM plus 10% aPRP is appropriate. For expansion, culture medium plus 5% aPRP is suitable. This protocol complies with GMP because of its xenogenic- and allogeneic-free medium components.

## Conclusion

UCB is a rich source of MSCs. UCB-MSCs can be isolated with xenogenic and allogeneic component-free medium. In this study, we successfully established a GMP-compliant UCB-MSC isolation protocol. Autologous aPRP can be used to replace FBS. Both aPRP and MNCs can be isolated from the same blood sample. In primary culture, MNCs should be cultured in IMDM plus 10% aPRP and 1% antibiotic-mycotic. However, in expansion culture, MSCs should be cultured in IMDM plus 5% aPRP and 1% antibiotic-mycotic. MSCs isolated by this protocol proliferate similarly as those in 10% FBS, maintain MSC phenotypes such as expression of CD13, CD44, CD73, CD90, CD105, CD106, and CD166, and do not express CD14, CD34, CD45, or HLA-DR. They also maintain their multilineage differentiation potential for adipocytes, osteoblast, and chondrocytes. In particular, the isolated MSCs do not form tumors at a high dose in athymic nude mice. This promising protocol is suitable for clinical applications of UCB-MSCs in the near future.

## Competing interests

The authors declare that they have no competing interests.

## Authors’ contributions

PVP, NBV conceived the study, performed PRP preparation, evaluated the effects of PRP on mesenchymal stem cell proliferation. VMP, NHT primarily cultured mesenchymal stem cells from mononuclear cells; TLBP, TTN collected umbilical cord blood, isolated mononuclear cells from umbilical cord blood; LTTD, ANTB carried out the differentiation assays; NKP evaluated the tumorigenecity of MSCs in mice model. All authors read and approved the final manuscript.
